# Prognosis of Upfront Surgery for Pancreatic Cancer: A Systematic Review and Meta-Analysis of Prospective Studies

**DOI:** 10.3389/fonc.2021.812102

**Published:** 2022-01-10

**Authors:** Nicolò Pecorelli, Alice W. Licinio, Giovanni Guarneri, Francesca Aleotti, Stefano Crippa, Michele Reni, Massimo Falconi, Gianpaolo Balzano

**Affiliations:** ^1^ Faculty of Medicine, Vita-Salute San Raffaele University, Milan, Italy; ^2^ Division of Pancreatic Surgery, Pancreas Translational and Clinical Research Center, San Raffaele Scientific Institute, Milan, Italy

**Keywords:** pancreatic neoplasms, upfront surgery, survival, adjuvant chemotherapy, neoadjuvant chemotherapy, pancreatic cancer

## Abstract

**Background:**

The rate of patients with pancreatic ductal adenocarcinoma (PDAC) receiving neoadjuvant chemotherapy is increasing, but upfront resection is still offered to most patients with resectable or borderline resectable disease. Encouraging data reported in adjuvant chemotherapy trials prompts surgeons towards upfront surgery, but such trials are subject to a significant selection bias. This systematic review aims to summarize available high-quality evidence regarding survival of patients treated with upfront surgery for PDAC.

**Methods:**

Pubmed, Cochrane, and Web of Science Databases were interrogated for prospective studies published between 2000 and 2021 that included at least a cohort of patients treated with upfront surgery for resectable or borderline resectable PDAC. The Cochrane Collaboration’s risk-of-bias tool for randomized trials (RoB-2) was used to assess risk of bias in all randomized studies. Patient weighted median overall survival (OS) and disease-free survival (DFS) were calculated.

**Results:**

Overall, 8,341 abstracts were screened, 17 reports were reviewed in full text, and finally 5 articles and 1 conference abstract underwent data extraction. Included studies were published between 2014 and 2021. All studies were RCTs comparing different neoadjuvant treatment strategies to upfront surgery. Three studies included only resectable PDAC patients, two studies recruited patients with resectable and borderline resectable disease, and one study selected only borderline resectable patients. A total of 439 patients were included in the upfront resection cohorts of the 6 studies, ranging between 20 to 180 patients per study. The weighted median OS after upfront surgery was 18.8 (95% CI 12.4 – 20.6) months. Median DFS was 9 (95% CI 1.6 – 12.5) months. Resection rate was 74.5% (range 65-90%). Adjuvant treatment was initiated in 68% (range 43-77%) of resected patients.

**Conclusions:**

High-quality data for PDAC patients undergoing upfront surgery is scarce. Meta-analysis from the included studies showed a significantly shorter OS and DFS compared to recently published studies focusing on adjuvant combination chemotherapy, suggesting that the latter may overestimate survival due to the exclusion of most patients scheduled for upfront surgery.

## Introduction

According to NCCN and Italian guidelines (1, 2), a neoadjuvant approach is suggested as the preferred treatment for borderline resectable tumors; for resectable tumors, either upfront surgery or neoadjuvant therapy may be considered. Upfront surgery is still, by far, the most frequently adopted approach for localized pancreatic cancer in both the USA and Europe, even though large retrospective studies (3, 4) and a few randomized controlled trials (RCTs) (5, 6) have observed prognostic improvements with neoadjuvant treatments. A recent paper analyzing national registries in the USA and in some European countries demonstrated that from 2014 to 2017, the rate of pancreatic cancer patients receiving preoperative chemotherapy was 27.6% in the USA, 4.9% in Germany, 7.0% in the Netherlands, and 3.4% in Sweden (7).

The low penetration of neoadjuvant therapy into the clinical setting can be partially attributed to the lack of robust evidence supporting it, with few adequately sized RCTs to date. Additionally, recent RCTs on adjuvant combination chemotherapies have found marked prognostic improvements after resection, supporting the surgery first approach: the ESPAC-4 trial reached a postoperative overall survival (OS) of 30 months with adjuvant gemcitabine and capecitabine (8), the APACT trial estimated an OS of 41.8 months with nab-paclitaxel + gemcitabine (9), and PRODIGE 24 estimated a remarkable OS of 54 months with adjuvant modified-FOLFIRINOX (10). The cohorts enrolled in such trials are however subject to a significant selection bias and the prognosis of candidates for upfront surgery (i.e. patients with resectable disease) is actually worse than what is described by trials on adjuvant therapies. Candidates for surgery include patients with occult metastases or unresectable tumor found at surgery, those who do not adequately recover from surgery to receive adjuvant treatments (i.e., patients experiencing severe postoperative complications or mortality), and patients who experience early tumor recurrence before starting adjuvant therapy. These patients may be considered resectable according to radiological definitions but are ultimately not resected and are therefore excluded from many studies. The consequence of this selection is that surgeons tend to overestimate the patients’ survival of upfront surgery.

The goal of this study was to analyze the existing literature and to define the prognosis of pancreatic cancer patients by the intention to treat with upfront surgery, as shown by prospective studies of adequate quality.

## Methods

This systematic review was performed according to PRISMA (Preferred Reporting Items for Systematic Reviews and Meta-Analyses) statement (11, 12). No specific funding was used to carry out this study. The literature was reviewed systematically by searching MEDLINE (Pubmed), Cochrane Library, and Web of Science (Scopus) for studies published between 1 January 2000 and 4 July 2021. The search strategy included the following keywords or Medical Subject Heading terms: ‘pancreatic neoplasms’, ‘survival’, ‘resectable’, ‘upfront surgery’ and ‘neoadjuvant’ which were combined with AND or OR. No language restrictions were used. The search was conducted to answer the research question: what are the outcomes of patients with resectable or borderline resectable pancreatic cancer patients undergoing upfront surgery?

### Inclusion and Exclusion Criteria

Studies were included in the review if they met the following criteria: (1) included adult patients with resectable or borderline resectable pancreatic cancer undergoing upfront surgery; (2) had a prospective design; (3) reported overall survival by intention to treat. No selection was made based on the type of adjuvant treatment. Conference abstracts satisfying inclusion criteria were included if reporting results of a completed study and primary outcome measure was reported. Studies reported as prospective but using an institutional database or registry that was not previously registered were excluded. Retrospective studies, review articles, notes, letters, case reports, animal studies, and studies that reported on only specific groups of patients were excluded.

### Outcome Measures

The primary outcome measure of this review was median overall survival (OS) in months. Additionally, 1-year OS rate and median disease-free survival (DFS) were extracted.

Secondary outcomes of interest included resection rate, reason for unresectability, pathological lymph-nodes rate, postoperative complications, and initiation of adjuvant treatment rate. Data on severe postoperative morbidity, type of adjuvant chemotherapy were also extracted.

### Study Selection and Data Extraction

Titles and abstracts yielded by the search strategy were screened for eligibility by two independent reviewers (N.P. and A.W.L.). Articles that were clearly irrelevant were excluded. The remaining full-text articles were then screened against the selection criteria by the same two reviewers. Disagreements were resolved by consensus within the research group.

Data were then extracted from the articles into a standardized data collection form. In addition to the outcome measures of interest, information about the study design, inclusion and exclusion criteria, number of patients and type of operation were collected.

### Quality Assessment

The Cochrane Collaboration’s risk-of-bias tool for randomized trials (RoB-2) (13) was used to assess risk of bias for all RCTs, including randomization process, deviations from intended interventions, missing outcome data, measurement of the outcome, and selection of the reported results. Each domain was classified as having a low, high, or some concerns of bias.

### Analysis

The weighted median OS and DFS and relative 95% confidence interval (CI) was calculated for all studies reporting this information. The weighted estimate of median survival was calculated using the formula suggested by Gillen and colleagues (14) in a previous systematic review:


$$m_{p}={\left(\sum_{i=1}^{k}\frac{w_{i}}{m_{i}}\right)}^{-1}$$

where *m_i_* stands for median survival in a study population *i*, which ranges from 1 to *k*, where *k* is the number of included studies and *w_i_* refers to a study-specific weight function. The number of study participants divided by the overall number of evaluable patients was used as the weight.

Descriptive statistics including median, and 95% confidence interval were reported for resection, R0 and initiation of adjuvant chemotherapy rate.

## Results

**Figure 1:**
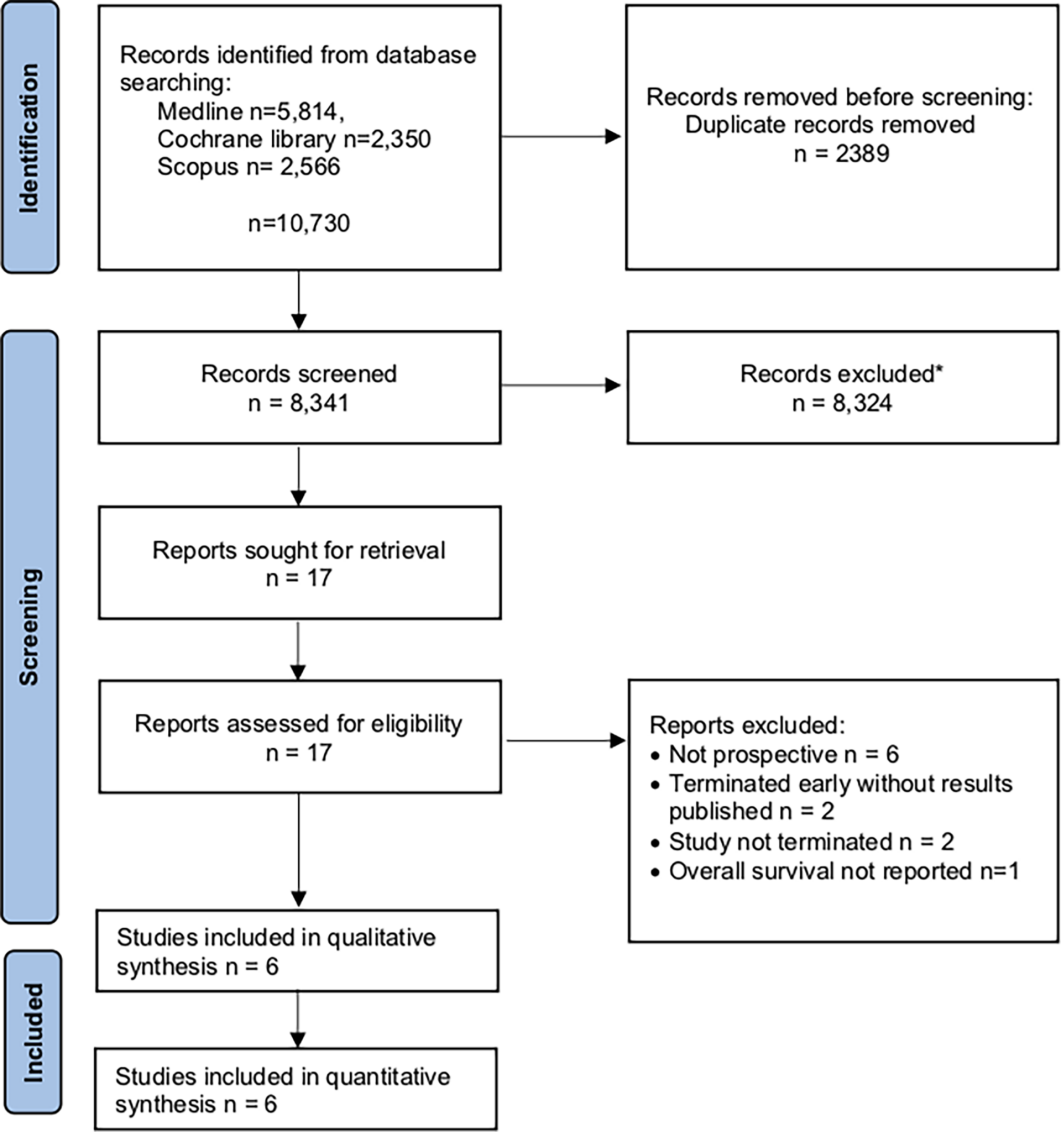
PRISMA flow chart showing selection of articles for review. *Excluded: animal studies, reviews, retrospective trials, locally advanced/metastatic disease.

The PRISMA flow chart is reported in **Figure 1**. Overall, 8,341 abstracts were screened, 17 reports were reviewed in full text, and finally 5 articles and 1 conference abstract underwent data extraction. Included studies were published between 2014 and 2020. The excluded articles and reasons for exclusion are provided as **Supplementary Material 1**. The main reason for exclusion was retrospective study design using non-previously registered databases.

### Characteristics of the Included Studies

**Table 1** reports the characteristics of included studies. All studies were RCTs comparing different neoadjuvant treatment strategies to upfront surgery for PDAC. Three studies were multi-center, while three were conducted in a single center. Three studies included only resectable PDAC patients, two studies recruited patients with resectable and borderline resectable disease, and one study selected only borderline resectable patients. All studies had planned an adjuvant chemotherapy treatment with a single drug except for one of the two cohorts from Reni et al. (17), where combination chemotherapy was planned. Patient recruitment for the studies spanned from 2003 to 2017. Notably, 2 studies were terminated early for slow patient accrual [Golcher et al. (15) and Casadei et al. (16)] and one study [Jang et al. (6)] was interrupted early by the safety monitoring committee because of the superiority of the neoadjuvant treatment group compared to upfront surgery. Studies inclusion and exclusion criteria are shown as **Supplementary Material 2**.

**Table 1:** Summary of characteristics of included studies.

Reference	Country	No. of patients	Years of recruitment	Study design	Comparative group	Tumor stage	Primary outcome	Tumor location	Adjuvant treatment planned
Golcher (15)	Germany	33	2003 - 2009	RCT	Neoadjuvant CT-RT	R	Overall Survival	Head: 33 (100%)	Gemcitabine
Casadei (16)	Italy	20	2007 - 2014	RCT	Neoadjuvant CT-RT	R	R0 resection	Head: 20 (100%)	Gemcitabine
Reni (17)	Italy	26	2010 - 2015	RCT - multicenter	Neoadjuvant CT	R	Event-free at 1 year	Head: 25 (96%)	Gemcitabine
								Body-tail: 1 (4%)	
		30						Head: 26 (87%)	PEXG
								Body-tail: 4 (13%)	
Jang (6)	Korea	23	2012 - 2014	RCT	Neoadjuvant CT-RT	BR	2-year survival rate	Head: 17 (74%)	Gemcitabine
								Body-tail: 6 (26%)	
Unno (18)	Japan	180	2013 - 2016	RCT - multicenter	Neoadjuvant CT	R, BR	Overall survival/Resection rate	Head: 130 (72.2%)	S-1
								Body-tail: 50 (27.8%)	
Versteijne (5)	Netherlands	127	2013 - 2017	RCT - multicenter	Neoadjuvant CT-RT	R (n=68), BR (n=59)	Overall survival	Head: 117 (92%)	Gemcitabine
								Body-tail: 10 (7.8%)	

Data are number of patients (%) referred to patients who underwent upfront surgery.

RCT, randomized controlled trial; CT, chemotherapy; RT, radiotherapy; R, resectable; BR, borderline resectable; PEXG, cisplatin, epirubicin, gemcitabine and capecitabine.

Methodological quality assessment of the included trials using the Cochrane’s RoB-2 tool is reported as **Supplementary Material 3**. The tool provided by Cochrane was utilized to assess the studies for risk of bias. The study by Reni et al. (19) was noted as having two patients who deviated from the intended interventions due to protocol violations. The study by Golcher et al. (15) did not have any statistical analysis plan available that was dated prior to the completion of the study. In the conference abstract and available documentation by Unno et al. (18) it was unclear if certain groups of patients were resected or not as it was reported that 49 patients were not resected in the upfront surgery group, but then in the subcategories for these patients it is then reported that 25 patients were not resected.

A total of 439 participants were included in the upfront resection cohorts of the 6 studies. Most studies had small cohorts, while only two included more than 100 patients. Median patient age ranged between 59 and 68 years. Pancreatic cancer was localized in the head of the pancreas in 82% of 448 patients, while the remaining patients presented with a neoplasm of the pancreatic body or tail.

### Primary Outcomes

**Figure 2:**
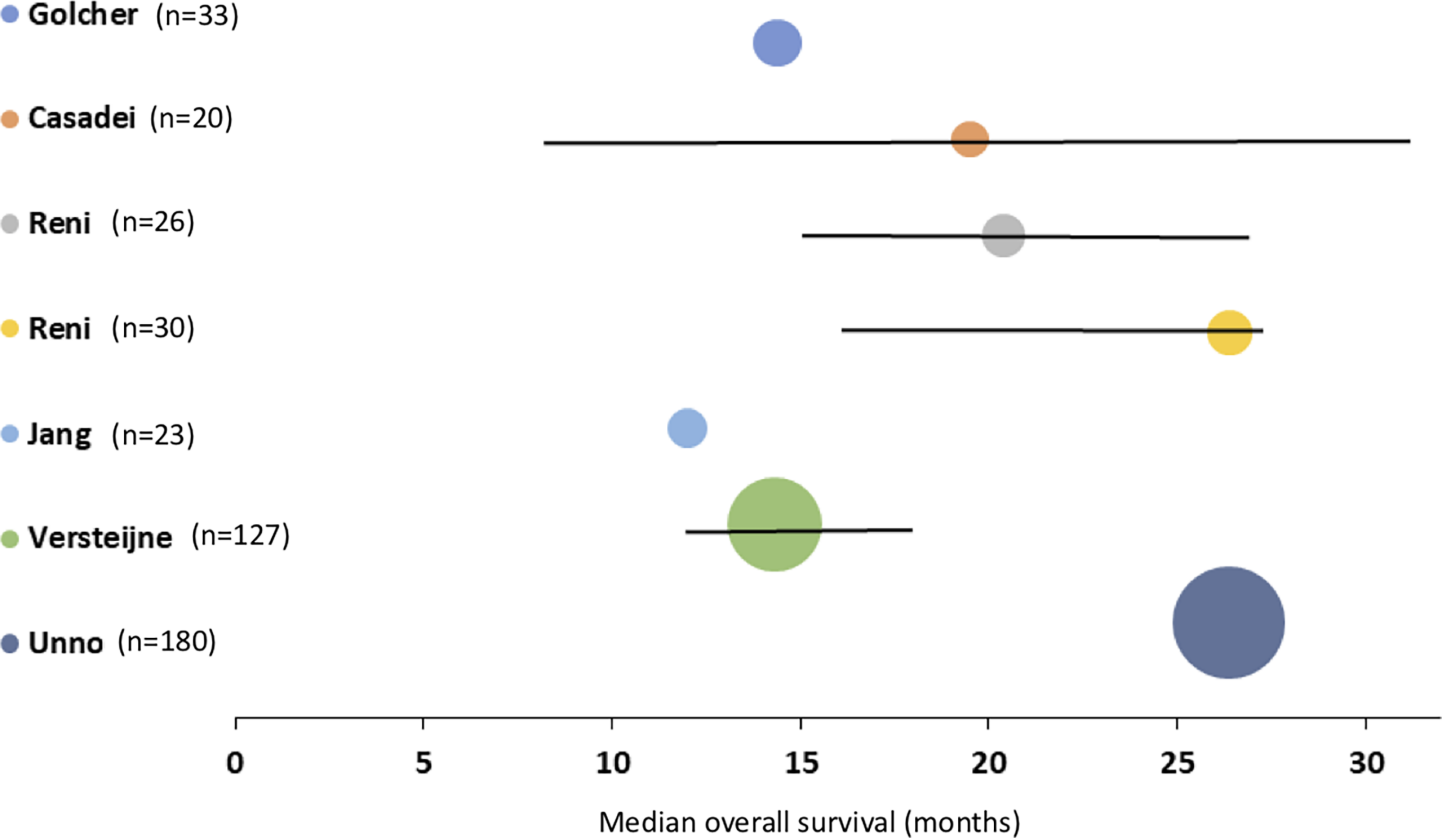
Median overall survival, with 95 per cent confidence intervals, for patients with pancreatic cancer after upfront surgery. The square of radius of the spheres is related to number of patients in the study.

Survival data are reported in **Table 2**. The weighted median OS by intention to treat after upfront surgery for PDAC was 18.8 (95% CI 12.4 – 20.6; range 12 - 26.6) months (**Figure 2**).

**Table 2:** Survival analysis in patients undergoing upfront surgery for pancreatic cancer.

Reference	No. of patients	Median age (years)	Median overall survival (months)	Median disease-free survival (months)	1-year overall survival
Golcher (15)	33	65.1	14.4	8.7	18 (55%)
Casadei (16)	20	67.5	19.5 (7.5 - 31.5)	n.r.	n.r.
Reni (17)	26	65	20.4 (14.6 - 25.8)	4.7 (0.9 - 8.9)	18 (69%)
	30	68	26.4 (15.8 - 26.7)	12.4 (5.4 - 19.4)	25 (83%)
Jang (6)	23	59	12	n.r.	12 (52%)
Unno (18)	180	66.0	26.6 (21.0 - 31.3)	11.3	135 (75%)
Versteijne (5)	127	67	14.3 (12.7 - 17.9)	7.7	76 (60%)
			R: 15.6	R: 9.3	
			BR: 13.2	BR: 6.2	

Data are reported as median (95% confidence interval) or number of patients (%).

n.r., not reported; R, resectable; BR, borderline resectable.

Overall survival rate at 12 months, as reported by four studies, was 62% of 239 patients (range 52% - 83%). The weighted median DFS reported by four studies including 396 patients was 9.0 (95% C.I. 1.6 – 12.5; range 4.7 – 12.4) months. Only two studies reported the outcomes of resected patients versus the whole cohort, impeding subgroup analyses.

### Secondary Outcomes

Secondary outcomes data are shown in **Table 3**. The overall resection rate was 74.5% (range 65-90%). Of the six studies only three reported reasons for not performing resective surgery, which mostly consisted of intraoperative evidence of metastatic or unresectable locally advanced disease. Additionally, R0 resection was achieved in 40.6% (range 27-70%) of 197 patients. Positive lymph-nodes were found in 75.6% (range 56-83%) of 197 patients.

**Table 3:** Resection, R0 rate and perioperative data after upfront surgery for pancreatic cancer.

Reference	No. of patients	Resection rate	Reason for unresectability	R0 rate	Patients with positive lymph nodes*	Overall postoperative complications	Severe postoperative complications	Adjuvant treatment initiated*
Golcher (15)	33	23 (70%)	10 n.r.	16 (70%)	13 (57%)	23/23 (100%) in resected	11/23 (47.8%) in resected	10 (43%)
						9/10 (90%) in non-resected	4 (40%) in non-resected	
Casadei (16)	20	15 (75%)	3 unresectable	5 (33%)	13 (87%)	11/20 (50%)	2 deaths (10%)	n.r.
			2 metastatic					
Reni (17)	26	22 (85%)	4 metastatic	6 (27%)	16 (73%)	15/22 (68.2%)	6/22 (27.3%)	17 (77%)
	30	27 (90%)	3 metastatic	10 (37%)	20 (74%)	18/27 (66.7%)	6/27 (22.2%)	20 (74%)
Jang (6)	23	18 (78%)	5 n.r.	6 (33%)	15 (83%)	12/18 (67%)	3/18 (16.7%)	13 (72%)
Unno (18)	180	129 (72%)	2 disease progression before surgery	n.r.	n.r.	n.r.	n.r.	n.r.
			49 n.r.					
Versteijne (5)	127	92 (72%)	4 disease progression before surgery;	37 (40%)	72 (78%)	46/92 (50%)	n.r.	65 (70%)
	R: 68	R: 54 (79%)	1 death before surgery	R: 32 (59%)		R: 31/68 (46%)		
	BR: 59	BR: 38 (64%)	1 refusal	BR: 5 (13%)		BR: 21/59 (36%)		
			15 unresectable					
			12 metastatic					
			2 unresectable + metastatic					

*Among patients who underwent resective surgery.

n.r., not reported; R, resectable; BR, borderline resectable.

Postoperative complications were frequent, occurring in 63% (range 50 - 100%) of 209 patients. Morbidity was classified as severe in 27% (range 10 - 47.8%) of 120 patients.

Adjuvant chemotherapy was initiated in 68% (range 43-77%) of 182 resected patients.

## Discussion

**Figure 3:**
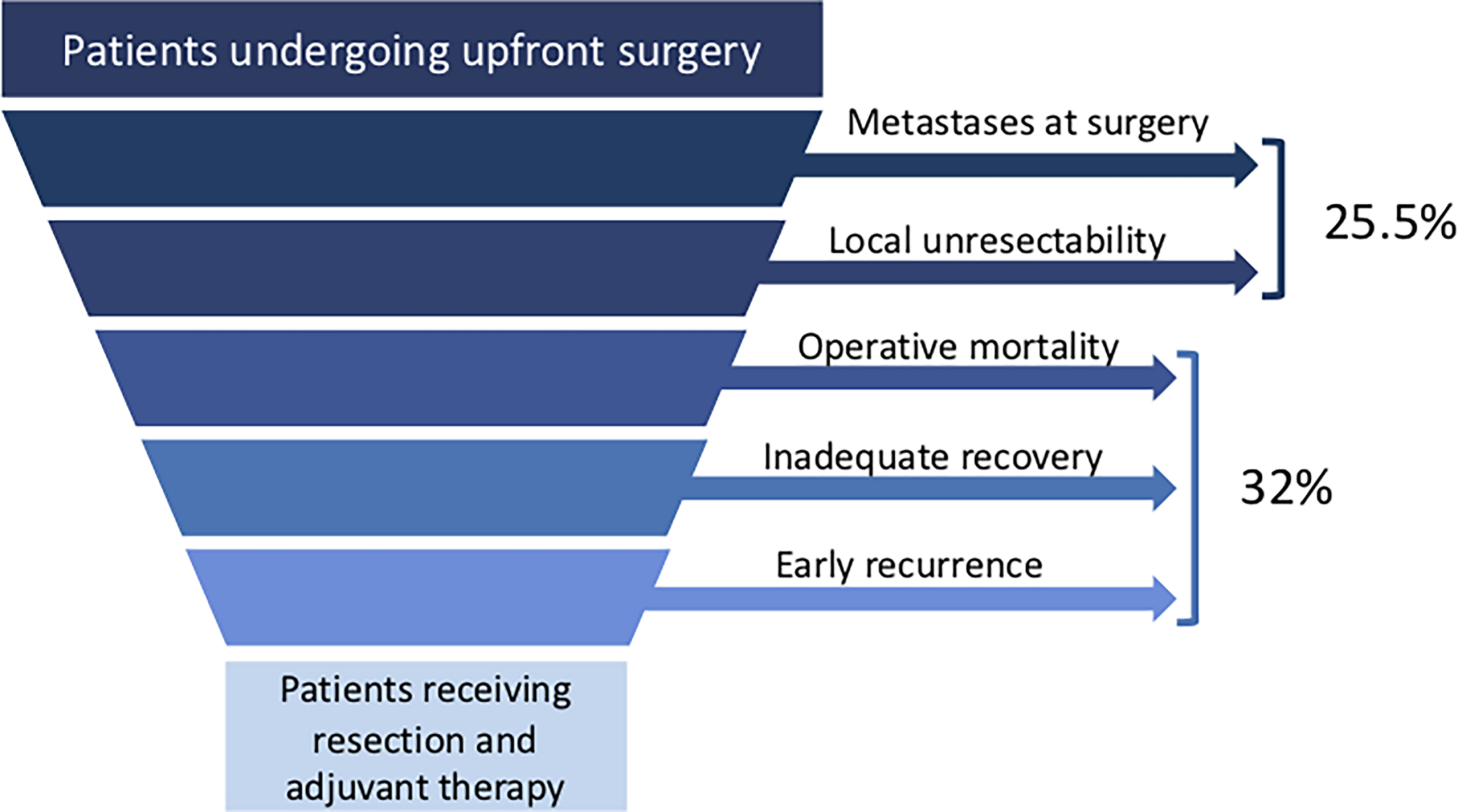
Patients candidate for upfront pancreatic cancer surgery and reasons for not completing the planned upfront resection followed by adjuvant chemotherapy. Values are referred to data resulting from this meta-analysis.

A frequent mistake surgeons make when discussing with patients that have resectable or borderline resectable pancreatic cancer amenable to radical resection is to overestimate their postoperative prognosis. This occurs because encouraging data on postoperative survival have been recently published by trials on adjuvant treatment. PRODIGE 24, a trial on modified adjuvant FOLFIRINOX for pancreatic cancer published in 2018, showed a median postoperative OS of almost 5 years (10). However, adjuvant studies enroll only a subset of the patients eligible for upfront surgery, as summarized in **Figure 3**. The PRODIGE 24 trial excluded the following categories of patients who are originally scheduled to undergo upfront surgery: patients who did not fully recover within 12 weeks after surgery (WHO performance >1); patients in which pancreatic cancer resection was not possible; those with postoperative early relapse, ascites, or pleural effusion or with CA19-9 levels >180 IU/ml before starting adjuvant therapy (10).

To contribute to the debate on neoadjuvant therapy use in resectable or borderline resectable pancreatic cancer, we performed a systematic review of the literature, looking for robust data on the real prognosis of candidates for upfront surgery. In the present study, a total of 439 candidates for upfront surgery were gathered from six prospective randomized controlled studies. The median OS of these patients was 18.8 (range 12-26.6) months, markedly lower than any study on adjuvant therapy of PDAC.

In our analysis we were able to retrieve the percentage of patients that would normally be excluded by adjuvant studies. The unresectability rate of patients undergoing immediate surgery was 26%. The largest multicenter trial included in our meta-analysis, PREOPANC, reported 28% of patients found to be unresectable (5). Of those patients who received resective surgery, one third (32%) failed to receive the subsequent planned adjuvant treatment. This figure can be further supported by a recent nationwide analysis in the Netherlands which found a similar rate (33%) of patients who did not ultimately receive adjuvant chemotherapy after immediate surgery (20). It has been suggested by a recent retrospective study on the U.S.A. National Cancer Database that neoadjuvant treatment could compensate for the prognostic disadvantage of failing to receive adjuvant therapy (21). Finally, when cancer is re-staged before starting adjuvant treatment, our study found that early recurrence is detected in about 10-20% of patients. Similarly, the APACT trial, which compares gemcitabine and gemcitabine combined with nab-paclitaxel in the adjuvant setting, excluded 17% of 1,226 screened patients because of early relapse suspicion (22). Also, the PACT-15 trial, which compares perioperative and adjuvant treatment, found that postoperative CT scan detected 12% early metastases in 49 patients undergoing upfront resection (17).

The effect on survival due to different selection criteria is also evident when comparing the prognosis of apparently similar treatment groups, such as in the PREOPANC and PRODIGE 24 trials. In both studies, the control arm consisted in adjuvant gemcitabine, with the same schedule. However, enrollment into the PREOPANC study occurred when a radical resection was predictable, selecting candidates eligible for upfront surgery. On the other hand, enrollment in the PRODIGE trial occurred after surgery, after re-staging, and after excluding patients with inadequate recovery. Owing to the difference in selection criteria, the median survival of PREOPANC patients in the adjuvant gemcitabine arm was 14.3 months, which was less than half of the survival observed in patients receiving adjuvant gemcitabine in the PRODIGE 24 trial (35.5 months) (23).

R0 is an important determinant of postoperative survival, as well as nodal involvement. In the present analysis, a radical resection was achieved in about 40% of resected patients, and negative lymph nodes were detected in 24.4% of patients undergoing upfront resection. Neoadjuvant treatment may improve such results: a recent systematic review reported 88.0% R0 rate and 52.5% N0 rate in patients receiving neoadjuvant FOLFIRINOX (24).

In the PREOPANC study, patients who received neoadjuvant therapy also had discouraging long-term outcomes, with a median survival of 16 months. This might be attributed to the inadequacy of the oncologic preoperative treatment, which was based on chemoradiotherapy (three courses of gemcitabine, associated with radiotherapy 36 Gy). Since most patients undergoing resection will experience distant failure (25, 26), it may be hypothesized that a more efficacious multiagent chemotherapy will offer better long-term results than chemoradiotherapy. In this context, PACT-15, a phase 2 randomized study that used multiagent chemotherapy PEXG, showed promising results in patients treated with the perioperative approach (3 months of chemotherapy before and 3 months after surgery). The median survival of 32 patients with resectable cancer randomized to the neoadjuvant approach was 38.2 months, with a 5-year survival in the per-protocol population of 50% (17).

The present systematic review has several limitations, mainly due to the small number of high-quality studies on ITT patients selected for an upfront surgery. We specified ITT as we wanted to collect outcomes of those patients who were offered immediate surgery but did not undergo resection for various reasons. It was decided to not include prospective studies on already resected upfront surgery patients to provide a realistic survival estimate for patients scheduled for immediate surgery. Trials reported as using prospective registries, but with a retrospective design analyzing data from non-previously registered databases were also excluded, limiting the number of patients included in this meta-analysis. The decision to possibly include conference abstracts of studies satisfying our selection criteria may be criticized, as it may carry the bias of unpublished data. However, only one recently published report was included in this review, as it reported the survival outcomes of interest. Further, most studies included in the review failed to report survival and outcome data for subgroups of patients of interest (e.g., resected versus non-resected patients; adjuvant vs. non adjuvant chemotherapy patients), preventing us from performing sensitivity analyses.

In conclusion, when facing a patient with a recent diagnosis of resectable or borderline resectable pancreatic cancer, the patient must be correctly informed about the expected survival of upfront surgery. Surgeons should be aware that it is not around 5 years, as reported by the PRODIGE 24 trial, but it is unfortunately around 19 months. This information will improve patients and surgeons’ awareness about the actual prognosis of localized pancreatic cancer and will allow to jointly select the adequate therapeutic option.

## Data Availability Statement

The original contributions presented in the study are included in the article/**Supplementary Material**, further inquiries can be directed to the corresponding author.

## Author Contributions

NP and GB designed the study. NP, AL, and GG reviewed and analyzed the data. NP, AL, and GB wrote the manuscript. NP, AL, GG, FA, SC, MR, MF, and GB reviewed the manuscript. All authors read and approved the final version of the manuscript.

## Funding

GG’s research fellowship, unrelated to this study, is funded by Fondazione Umberto Veronesi not-for profit organization.

## Conflict of Interest

The authors declare that the research was conducted in the absence of any commercial or financial relationships that could be construed as a potential conflict of interest.

## Publisher’s Note

All claims expressed in this article are solely those of the authors and do not necessarily represent those of their affiliated organizations, or those of the publisher, the editors and the reviewers. Any product that may be evaluated in this article, or claim that may be made by its manufacturer, is not guaranteed or endorsed by the publisher.
