# Supplementary material for: Prognosis of Upfront Surgery for Pancreatic Cancer: A Systematic Review and Meta-Analysis of Prospective Studies
Source: Front Oncol. 2022 Jan 10;11:812102. doi: 10.3389/fonc.2021.812102 (PMC8784375; doi:10.3389/fonc.2021.812102)
Supplement: Supplementary file 1 [file DataSheet_1.docx]

**Supplementary material**

**Supplementary material 1.** Studies excluded after full text review.

**Supplementary material 2.** Inclusion and exclusion criteria of studies included in this review.

**Supplementary material 3.** Cochrane Risk of Bias Tools Table

**Supplementary material 1.** Studies excluded after full text review

Reasons for exclusion:

- Retrospective study design using non-prospectively registered databases (n=6)

1. Bao P, Potter D, Eisenberg DP, Lenzner D, Zeh HJ, Lee lii KK et al. Validation of a prediction rule to maximize curative (R0) resection of early-stage pancreatic adenocarcinoma. HPB (Oxford) 2009; 11: 606–611.
2. Raptis DA, Fessas C, Belasyse-Smith P, Kurzawinski TR. Clinical presentation and waiting time targets do not affect prognosis in patients with pancreatic cancer. Surgeon 2010; 8: 239–246.
3. Tzeng CW, Tran Cao HS, Lee JE, Pisters PW, Varadhachary GR, Wolff RA et al. Treatment sequencing for resectable pancreatic cancer: influence of early metastases and surgical complications on multimodality therapy completion and survival. J Gastrointest Surg 2014; 18: 16–24.
4. Fujii T, Satoi S, Yamada S, Murotani K, Yanagimoto H, Takami H et al. Clinical benefits of neoadjuvant chemoradiotherapy for adenocarcinoma of the pancreatic head: an observational study using inverse probability of treatment weighting. J Gastroenterol 2017; 52: 81–93
5. Fujii T, Yamada S, Murotani K, Kanda M, Sugimoto H, Nakao A et al. Inverse probability of treatment weighting analysis of upfrontsurgery versus neoadjuvant chemoradiotherapy followed by surgery for pancreatic adenocarcinoma with arterial abutment. Medicine (Baltimore) 2015; 94: e1647.
6. Skau Rasmussen L, Vittrup B, Ladekarl M, Pfeiffer P, Karen Yilmaz M, Østergaard Poulsen L, Østerlind K, Palnæs Hansen C, Bau Mortensen M, Viborg Mortensen F, Sall M, Detlefsen S, Bøgsted M, Wilki Fristrup C. The effect of postoperative gemcitabine on overall survival in patients with resected pancreatic cancer: A nationwide population-based Danish register study. Acta Oncol. 2019 Jun;58(6):864-871.

- Not reporting overall survival data (n=1)

1. P Ghaneh, D Palmer, S Cicconi, C Halloran, E Psarelli, C Rawcliffe, R Sripadam, S Mukherjee, J Wadsley, A Al-Mukhtar, L Jiao, H Wasan, R Carter, J Graham, F Ammad, J Evans, C Tjaden, T Hackert, Bϋchler, J Neoptolemos for the European Study Group for Pancreatic Cancer (ESPAC). ESPAC-5F: Four arm, prospective, multicentre, international randomised phase II trial of immediate surgery compared with neoadjuvant gemcitabine plus capecitabine (GEMCAP) or FOLFIRINOX or chemoradiotherapy (CRT) in patients with borderline resectable pancreatic cancer. Paper presented online at: Annual National Study Session hosted by Pancreatic Cancer UK. 2020 October 8^th^.

- Early termination of the study, unpublished data (n=2)

1. Heinrich S, Pestalozzi B, Lesurtel M, Berrevoet F, Laurent S, Delpero JR, Raoul JL, Bachellier P, Dufour P, Moehler M, Weber A, Lang H, Rogiers X, Clavien PA. Adjuvant gemcitabine versus NEOadjuvant gemcitabine/oxaliplatin plus adjuvant gemcitabine in resectable pancreatic cancer: a randomized multicenter phase III study (NEOPAC study). BMC Cancer. 2011 Aug 10;11:346. doi: 10.1186/1471-2407-11-346. PMID: 21831266; PMCID: PMC3176241.
2. Tachezy M, Gebauer F, Petersen C, Arnold D, Trepel M, Wegscheider K, Schafhausen P, Bockhorn M, Izbicki JR, Yekebas E. Sequential neoadjuvant chemoradiotherapy (CRT) followed by curative surgery vs. primary surgery alone for resectable, non-metastasized pancreatic adenocarcinoma: NEOPA- a randomized multicenter phase III study (NCT01900327, DRKS00003893, ISRCTN82191749). BMC Cancer. 2014 Jun 7;14:411. doi: 10.1186/1471-2407-14-411. PMID: 24906700; PMCID: PMC4057592.

- Study is still active and recruiting patients (n=2)

1. Labori KJ, Lassen K, Hoem D, Grønbech JE, Søreide JA, Mortensen K, Smaaland R, Sorbye H, Verbeke C, Dueland S. Neoadjuvant chemotherapy versus surgery first for resectable pancreatic cancer (Norwegian Pancreatic Cancer Trial - 1 (NorPACT-1)) - study protocol for a national multicentre randomized controlled trial. BMC Surg. 2017 Aug 25;17(1):94. doi: 10.1186/s12893-017-0291-1. PMID: 28841916; PMCID: PMC6389186.
2. [Randomized multicenter phase II/III study with adjuvant gemcitabine versus neoadjuvant/adjuvant FOLFIRINOX in resectable pancreatic cancer: The NEPAFOX trial.](https://ascopubs.org/doi/abs/10.1200/JCO.2021.39.3_suppl.406) Salah-Eddin Al-Batran, Alexander Reichart, Ulli Simone Bankstahl, Claudia Pauligk, Thomas Werner Kraus, Wolf Otto Bechstein, Jorg Trojan, Matthias Behrend, Jochem Potenberg, Nils Homann, Marino Venerito, Wolfram Bohle, Michael Varvenne, Claus Bolling, Dirk M. Behringer, Karsten Kratz-Alber, Gabriele Margareta Siegler, Wael Hozaeel, and Thorsten Oliver Goetze. Journal of Clinical Oncology 2021 39:3_suppl, 406-406

**Supplementary material 2.** Inclusion and exclusion criteria of studies included in this review.

| Reference | Inclusion Criteria | Exclusion Criteria |
| --- | --- | --- |
| Golcher, 2014 | 1. Age 18-75 years 2. Histologically confirmed ductal adenocarcinoma of the pancreatic head 3. No infiltration of extra-pancreatic organs with the exception of the duodenum 4. Pancreatic tumor confirmed by high-resolution spiral CT (layer thickness preferably 3 mm) that is classified as resectable or probably resectable (vascular involvement ≤ 180° of one of the peripancreatic major vessels) 5. No distant metastasis or peritoneal spread 6. Karnofsky index ≥ 70 | 1. Tumor-specific prior treatment 2. Recurrent tumor 3. Prior or synchronous malignancy 4. Liver cirrhosis with platelets < 100,000/mm^3^ or PTT < 70% 5. Serum creatinine > 1.5 mg/dl, creatinine clearance < 70 ml/min (24h collection phase) 6. Severe cardio-pulmonary concomitant disease or other serious disease 7. HIV infection 8. Pregnancy or desire for children in female patients 9. Justified doubt as to the understanding or contractual capacity of the patient |
| Casadei, 2015 | 1. Age 18-80 years 2. Medical history without previous pancreatic resection or pancreatic cancer 3. ECOG = 0-1 4. ASA score < 4 5. Good renal, hepatic, cardiac, and hematological functions 6. Histologically proven resectable pancreatic adenocarcinoma | 1. Chemoradiotherapy in the past 6 months 2. Other neoplastic diseases diagnosed in the past 5 years 3. Major surgery, biopsy, or traumatic event in the past 28 days 4. HIV infection |
| Reni, 2018 | 1. Age 18-75 years 2. Previously untreated pancreatic adenocarcinoma 3. Karnofsky performance status > 60 4. Pathologically confirmed pancreatic ductal adenocarcinoma 5. Clinical stage I-II (TNM 2010) 6. Resectable disease (defined as the absence of invasion of the mesenteric artery or vein, portal vein, coeliac artery or hepatic artery) 7. Adequate bone marrow, liver and kidney function | 1. Personal history of other previous or concurrent malignancies at other sites 2. Pregnancy and lactation 3. Symptomatic duodenal stenosis 4. Concurrent treatment with other experimental drugs 5. Any physiological, familiar, sociological, or geographic conditions that can potentially interfere with adherence to the protocol or to follow-up |
| Jang, 2018 | 1. Age 18 - 75 years 2. Radiologic evidence of BRPC (NCCN guidelines, 2012) - MDCT 3. Histologically or cytologically proven pancreatic cancer 4. No history of previous chemoradiation therapy 5. Adequate bone marrow, hepatic, and renal function according to laboratory tests | 1. Undergone concomitant unplanned antitumor therapy (e.g. chemotherapy, radiotherapy, immunotherapy) 2. Had a concomitant or previous malignancy (except cancer that had been in complete remission for > 5 years) 3. Had uncontrolled systemic disease (e.g. infectious disease and cardiovascular disease) |
| Versteijne, 2020 | 1. Histologically or cytologically confirmed adenocarcinoma of the pancreas 2. Primarily resectable or borderline resectable tumors 3. Ability to undergo surgery and chemotherapy (normal blood count and adequate renal function) | 1. T1 resectable tumors, locally advanced, unresectable tumors or distant metastases 2. Cytologically proven N2 lymph node metastases 3. Previous active malignancy shorter than 5 years before diagnosis of pancreatic cancer or co-morbidity or previous treatment precluding surgery or radiochemotherapy |
| Unno, 2019 | 1. Age 20-79 years 2. Treatment-naïve PDAC with histological or cytological diagnosis 3. Localized tumor without distant metastasis confirmed by radiological evaluation (contrast enhanced CT scan) 4. R0/1 resectable, without arterial abutment 5. Can tolerate curative surgery 6. ECOG 0-1 7. Spared organ function 8. Adequate oral intake | 1. Pulmonary fibrosis or interstitial pneumonia 2. Severe diarrhea 3. Synchronous malignancy except 4. Metachronous malignancy except for disease having ≥ 3 relapse-free survivals 5. Active infection 6. Regular use of frucitocin, phenytoin or warfarin 7. Pregnancy, breastfeeding or desire of a woman to preserve fertility |

**Supplementary material 3.** Cochrane Risk of Bias Tools Table

**
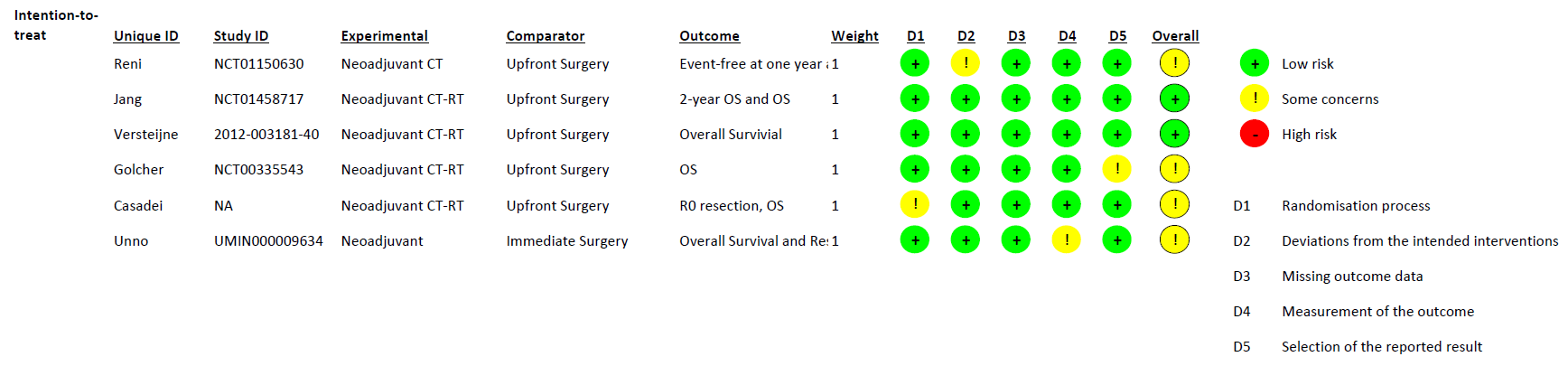
**
